# Axial length acquisition success rates and agreement of two swept-source optical biometers in eyes with dense cataracts

**DOI:** 10.3389/fmed.2024.1449867

**Published:** 2024-09-25

**Authors:** Paz Orts-Vila, Santiago Tañá-Sanz, Cristina Tello-Elordi, Robert Montés-Micó, Pedro Tañá-Rivero

**Affiliations:** ^1^Oftalvist, Alicante, Spain; ^2^Department of Optics and Optometry and Vision Sciences, University of Valencia, Valencia, Spain

**Keywords:** cataracts, biometry, optical, dense, optical coherence tomography

## Abstract

**Background:**

Swept-source optical coherence tomography–based (SS-OCT) biometers have been used in different clinical studies with the aim of assessing the accuracy of the technique, specifically in eyes with dense cataracts. Our objective is to evaluate the axial length acquisition success rates and agreement of two SS-OCT biometers when measuring axial length and biometric parameters in eyes with dense cataracts.

**Methods:**

46 eyes (46 patients) with dense cataracts (LOCS III grade ≥ 4) were measured 3 consecutive times using the Eyestar 900 and Argos SS-OCT biometers. Keratometry (K1, flat and K2, steep), central corneal thickness (CCT), white-to-white (WTW), anterior-chamber-depth (ACD), lens-thickness (LT), and axial length were measured using both biometers. The percentage acquisition success rate and a Bland–Altman analysis to determine the agreement between the biometers were calculated. Corrected and uncorrected distance visual acuity, subjective refraction, and axial length (to assess preoperative axial length accuracy) were measured 1-month post-cataract surgery.

**Results:**

The mean LOCS III score was 4.37 ± 0.68. The acquisition success rates for both biometers was 100%. There were statistically significant differences between the two SS-OCT biometers for all parameters evaluated (*p* < 0.05). The mean differences for K1, K2, CCT, WTW, ACD, LT and axial length were 0.106 D, 0.128 D, −6.347 μm, −0.054 mm, 0.095 mm, 0.110 mm, and −0.036 mm, respectively. The mean pre- and post-surgery axial length difference was −0.036 mm for the Eyestar 900 and −0.020 mm for the Argos. This difference was ≤ 0.1 mm in 97.82% of eyes with the Eyestar 900 and in 100% of eyes with the Argos.

**Conclusion:**

SS-OCT biometry successfully measures axial length in dense cataracts. The differences between biometers in some parameters may have a clinically significant impact but should be judged individually. The pre- and post-surgery axial length differences for each biometer can be considered clinically negligible and should not affect the IOL power calculation.

## 1 Introduction

In recent years, various optical technologies have been developed and applied to ocular biometry. The goal of these techniques is to accurately measure the different ocular parameters, with axial length being the most important when calculating the intraocular lens (IOL) power in cataract surgery. Partial coherence interferometry (PCI), optical low-coherence reflectometry (OLCR) and, more recently, swept-source optical coherence tomography (SS-OCT) have been used worldwide and show good outcomes. Failure rates of about 35–38% have been reported, with increased severity of posterior subcapsular cataracts with PCI and OLCR (1), although a new axial measurement mode using OLCR in eyes with a dense cataract has significantly reduced the failure rate to 1.6% (2). New optical biometers based on SS-OCT are more frequently successful at measuring axial length in dense cataracts and when these fail, the cataract type is mainly mature white or grade 4 or above (3). The lack of light penetrating to the retina or the patient’s inability to fixate during the biometric measurement may play a significant role in this failure. The use of Fourier-domain OCT, allowing better penetration, may be considered the main difference between the SS-OCT and PCI technologies that improves the axial length measurement success in eyes with dense opacity (4).

Established SS-OCT biometers have been used in different clinical studies with the aim of assessing the accuracy of the technique, specifically in eyes with dense cataracts (5–11). A recent SS-OCT biometer, Eyestar 900 (Haag-Streit Holding AG, Koeniz, Switzerland), has been put on the market and evaluated in cataract, healthy and keratoconic eyes (12–17). Those studies showed that this biometer produces highly repeatable measurements that agree well with other SS-OCT- and OLCR-based biometers. However, there is no data in the literature as to whether the new biometer effectively measures eyes with dense cataracts, nor its agreement with other SS-OCT, such as the Argos (Alcon Labs, Fort Worth, USA), in this type of eyes.

The purpose of this clinical study was therefore to assess axial length acquisition success rates and post-refractive accuracy, and to compare the measurements of keratometry (K1: flattest keratometry; K2: steepest keratometry), central corneal thickness (CCT), white-to-white distance (WTW), anterior chamber depth (ACD), lens thickness (LT), and axial length in eyes with dense cataracts obtained using the Eyestar 900 and the Argos SS-OCT biometers.

## 2 Materials and methods

This was a single-center, prospective, comparative study carried out in the Oftalvist clinical center (Alicante, Spain). It followed the tenets of the Declaration of Helsinki with all patients providing written informed consent prior to being enrolled in the study. The study was approved by the Ethics Committee of Hospital Clínico San Carlos (Madrid, Spain, number 23/621-O_P) and registered in the public German Clinical Trials Registry prior to the begin of the study (identifier: DRKS00032844).

### 2.1 SS-OCT biometers

The Eyestar 900 is a fully automated device that performs automatic centration and measurement using a wavelength of 1,060 nm with a scan speed of 30 kHz for axial length measurements (range from 14 to 38 mm) and is group refractive index based biometer using a single refractive index to calculate axial length (version 1.6). It uses an infrared light-emitting diode (LED) source of 850 nm to measure 32 points to provide dual zone keratometry. The Argos uses a wavelength of 1,060 nm (20 nm bandwidth) measuring at a rate of 3000 A-scans/sec, and is a sum-of-segments biometer based that uses different refractive indices to calculate axial length (version 2.4.0). Keratometry, using a 1.3375 corneal refraction index, is measured from the OCT image in conjunction with a 2.2 mm diameter ring made up of 16 LEDs; optical distances are measured using the OCT taking into account different refractive indices (cornea: 1.376; aqueous and vitreous humors: 1.336; lens: 1.410). It has an enhanced retinal visualization (ERV) mode in which the optical path length is measured by minimizing the effect of attenuation and by changing the OCT sensitive position to the retinal side. By combining this optical path length with the anterior segment information up to the posterior surface of the crystalline lens, measured using the standard mode, the axial length in ERV mode is calculated.

### 2.2 Patients and procedure

All patients included in the study underwent a full ophthalmological examination, including preoperative logMAR corrected distance visual acuity (CDVA), subjective refraction, and slit-lamp and dilated fundus examinations. The inclusion criteria were eyes with a lens opacities classification system (LOCS) III (18) grade of ≥ 4 for nuclear, cortical or posterior subcapsular cataracts. The exclusion criteria included patients with other ocular co-morbidities (keratoconus, glaucoma, dry eye, diabetic retinopathy, uveitis, retinal detachment, or prior refractive surgery), patients with systemic disease or systemic medications that could affect visual acuity or refraction, ocular trauma, poor fixation, no axial length measurement with any of the devices, and previous continuous contact lens wear. Post-refractive surgery eyes were not included since they can be affected by inaccurate measurement of keratometric values, and the inclusion of these eyes could generate a bias of data analysis.

Ocular biometry was measured three times, in a random order, with each biometer before pupil dilation since mydriasis can affect biometry. Note that axial length is not influenced by mydriasis but other parameters yes (19). If the axial length could not be measured with the Argos biometer in the standard mode, the ERV mode was used. Only one eye from each patient was used for the data analysis (in cases where both eyes could be included, the eye to be included was chosen randomly) and both biometers were calibrated prior to start the measurements following manual instructions. For the Argos device the system captures a reference image for noise suppression and uses a calibration tool. After the system is ready for measurement, the software asks the user to insert the calibration tool in the relief placed on top of the forehead rest. Then the software shows an alignment window composed of a complementary metal oxide semiconductor (CMOS) image and an OCT image, and the operator aligns laterally and axially the LED ring projection inside the tracking zone using the joystick until proper alignment is achieved. The system automatically calculates the function check results. If the result is satisfactory, the software will move to the “patient entry” window. For the Eyestar 900 calibration was done directly by the company at the clinic. K1, K2, CCT, WTW, ACD, LT, and axial length parameters were recorded using the two SS-OCT biometers. LogMAR CDVA, and uncorrected distance visual acuity (UDVA), and subjective refraction were measured 1 month post-cataract surgery (phacoemulsification). To assess the accuracy of the preoperative axial length, pre- and postoperative axial length measurements were compared in eyes in which the axial length was successfully measured using both SS-OCT biometers.

### 2.3 Statistical analysis and sample size calculation

Axial length acquisition success rates were calculated as percentages and other variables as the mean, standard deviation (SD), and minimum and maximum values. The McNemar test was used to compare the acquisition rate between the two SS-OCT biometers. In relation to the other variables (K1, K2, CCT, WTW, ACD, LT, and axial length), the normality distribution was checked using the Shapiro–Wilk test using the SPSS software (IBM Corp., USA). The statistically significant differences between the measurements taken with the two arms were evaluated using the paired *t*-test (if normality was met; otherwise, the Wilcoxon Sign Rank test was used). A *p*-value of < 0.05 was considered statistically significant. In addition, the agreement between the two optical biometers was assessed by applying a Bland–Altman analysis. The average difference, the confidence interval of the average difference at 95%, and 95% limits of agreement (LoA, calculated as the mean difference ± 1.96 SD) were also ascertained. In addition, pre- and postoperative axial length values in the same eye were also compared by means of the paired *t*-test (if normality was met; otherwise, the Wilcoxon Sign Rank test was used). Bland-Altman plots were also used to examine possible differences between the pre- and postoperative axial length measured using the two SS-OCT biometers.

Considering a type I error of 5%, two-tailed hypothesis, 90% power, P1 = 93.4% [this refers to the Argos acquisition rate from a previous study (9)], P2 = 61.5% {this refers to the Eyestar 900 acquisition rate being similar to another SS-OCT biometer: the IOLMaster 700 [Carl Zeiss Meditec AG, Jena, Germany (13)]} and a drop-out rate of 10%, the sample included 46 eyes. The sample size was computed using PASS 2023, version 23.0.2 (NCSS, LLC, Kaysville, Utah, USA).

## 3 Results

Forty-six eyes from 46 patients (28 females) with dense cataracts were analyzed in our study. The mean and SD of LOCS III for the study sample was 4.37 ± 0.68, ranging from 4 to 6. There were 34 (73.9%), 7 (15.2%), and 5 eyes (10.9 %) with grades 4, 5 and 6, respectively, among the subjects. The mean age of the patients was 70.85 ± 10.94 years (mean ± SD). Note that no adverse events were reported over the entire duration of the study. Specifically, the mean spherical equivalent of the eyes in our sample was −1.08 ± 3.97 D, with a range from −14.00 to 4.25 D, and the preoperative CDVA was 0.32 ± 0.30 logMAR. Table 1 shows the mean values, SD, ranges and 95% confidence interval for the different parameters obtained using the two SS-OCT biometers. There were statistically significant differences between the two devices in the results for K1, K2, CCT, WTW, ACD, and axial length (*p* < 0.05). The mean UDVA and CDVA 1 month post-surgery were 0.08 ± 0.11 logMAR (range from −0.10 to 0.50) and −0.01 ± 0.03 logMAR (range from −0.10 to 0.06), respectively. At this follow-up time, the mean spherical equivalent was −0.11 ± 0.42 D (range from −1.00 to 1.63), with 86.95% of eyes being within ± 0.50 D and 97.85% within ± 1.00 D of the target refraction. The mean axial length 1 month after the surgery was 23.77 ± 1.46 mm (range from 21.28 to 27.68) and 23.79 ± 1.50 (range from 21.20 to 27.81) mm, for the Eyestar 900 and Argos SS-OCT biometers, respectively. There were statistically significant differences between pre- and post-surgery values (*p* < 0.001) for the two biometers.

**TABLE 1:** Mean ± standard deviation (range) [95% confidence interval] of the different parameters examined for the two optical biometers.

Parameter	Eyestar 900	Argos	*p*-value
K1 (D)	43.33 ± 1.17	43.44 ± 1.20	< 0.001*
	(40.45–46.01)	(40.54–46.17)	
	[43.00–43.67]	[43.10–43.79]	
K2 (D)	44.52 ± 1.22	44.65 ± 1.28	< 0.001*
	(41.61–47.67)	(41.71–47.89)	
	[44.17–44.87]	[44.28–45.02]	
CCT (μm)	549.39 ± 32.48	543.04 ± 31.77	< 0.001*
	(494–631)	(484–610)	
	[540.01–558.78]	[533.86–552.22]	
WTW (mm)	11.99 ± 0.41	11.93 ± 0.39	< 0.001*
	(11.19–13.06)	(11.15–12.80)	
	[11.87–12.11]	[11.82–12.05]	
ACD (mm)	3.15 ± 0.50	3.25 ± 0.49	< 0.001*
	(2.02–4.14)	(2.18–4.23)	
	[3.01–3.30]	[3.10–3.39]	
LT (mm)	4.49 ± 0.51	4.60 ± 0.51	< 0.001*
	(3.00–5.78)	(3.15–5.92)	
	[4.35–4.54]	[4.46–4.75]	
AL (mm)	23.83 ± 1.51	23.79 ± 1.46	< 0.001*
	(21.24–27.93)	(21.28–27.76)	
	[23.39–24.26]	[23.37–24.21]	

K, keratometry; CCT, central corneal thickness; WTW, white-to-white distance; ACD, anterior chamber depth; LT, lens thickness; AL, axial length;

*significant differences < 0.05.

**FIGURE 1:**
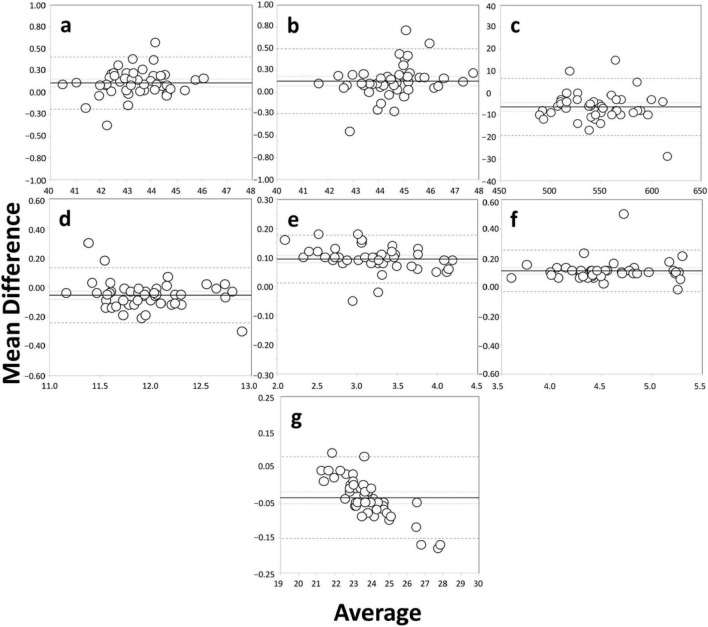
Bland–Altman plots of the mean difference versus the average of K1 [flattest keratometry, D, **(a)**], K2 [steepest keratometry, D, **(b)**], CCT [central corneal thickness, μm, **(c)**], WTW [white-to-white, mm, **(d)**], distance, ACD [anterior chamber depth, mm, **(e)**], LT [lens thickness, mm, **(f)**], and axial length [mm, **(g)**] used to compare the two SS-OCT optical biometers. The plots show the mean (continuous line), lower and upper limits of agreement (± 1.96 SD [standard deviation], peripheral dotted lines), and the lower and upper confidence intervals (95%).

**FIGURE 2:**
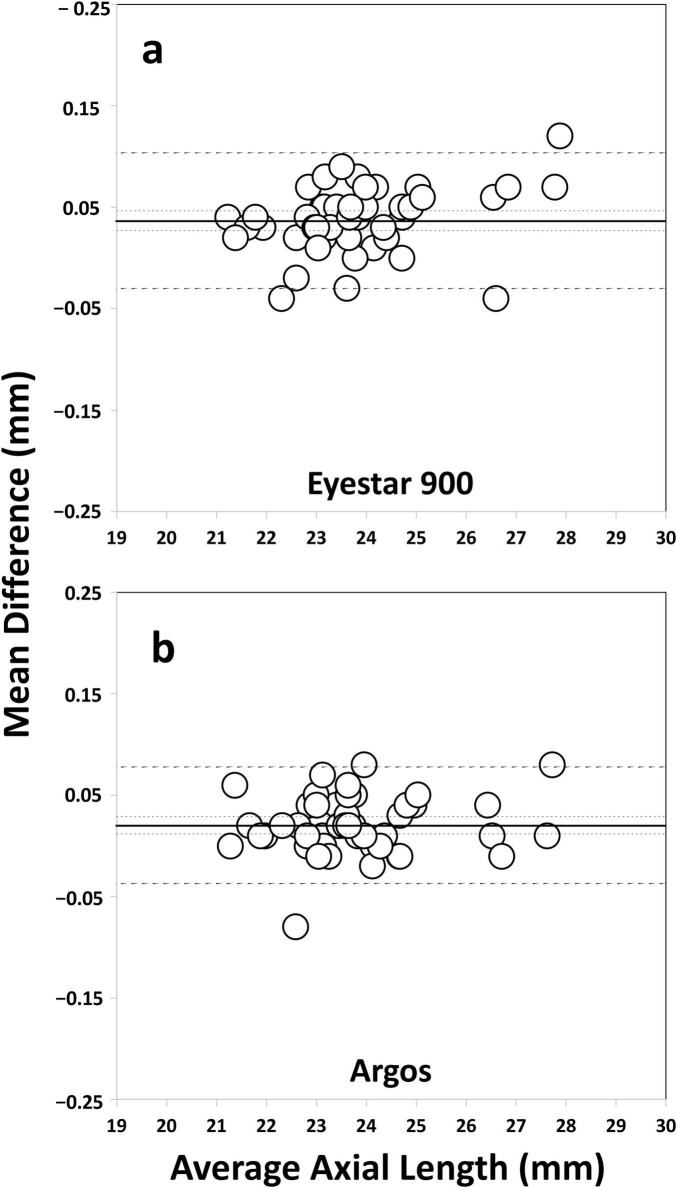
Bland–Altman plots of the mean difference versus the average axial length (mm) used to compare the pre- and postoperative values measured using the Eyestar 900 **(a)** and Argos **(b)** SS-OCT optical biometers. The plots show the mean (continuous line), lower and upper limits of agreement (± 1.96 SD [standard deviation], peripheral dotted lines), and the lower and upper confidence intervals (95%).

The axial length acquisition success rate was 100% (46 eyes) for both SS-OCT biometers. ERV mode of the Argos biometer were not used since all measurements were possible using the Eyestar 900 and Argos in the standard mode. Table 2 shows the level of agreement between the parameters obtained using the two SS-OCT biometers, with a mean difference of ± SD, 95% confidence interval, 95% LoA, and the LoA width for all pairwise comparisons. The Bland–Altman plots presented in Figure 1 are shown in several graphs to highlight the differences in K1 (a), K2 (b), CCT (c), WTW (d), ACD (e), LT (f), and axial length (g) in the two SS-OCT biometers. The agreement of the axial length measured pre- and postoperatively was also analyzed using Bland-Altman plots for the whole sample. Figure 2 using the Eyestar 900 (a) and Argos (b) SS-OCT biometers. In this case the mean difference ± SD, 95% confidence interval, 95% LoA, and the LoA were 0.036 ± 0.034, 0.027 to 0.046, −0.030 to 0.103, and 0.134 mm for the Eyestar 900, and 0.020 ± 0.029, 0.012 to 0.029, −0.037 to 0.078, and 0.115 mm for the Argos. The difference between the pre- and post-surgery axial length was ≤ 0.1 mm in 97.82% of eyes (*n* = 45) using the Eyestar 900 and in 100% of eyes (*n* = 46) with the Argos.

**TABLE 2:** Agreement between the two biometers for the different parameters examined.

Parameter	Mean difference ± SD	95% CI	95% LoA	LoA Width
K1 (D)	0.106 ± 0.152	0.063, 0.151	−0.191, 0.405	0.596
K2 (D)	0.128 ± 0.189	0.074, 0.184	−0.243, 0.500	0.744
CCT (μm)	−6.347 ± 6.630	−8.264, −4.432	−19.344, 6.648	25.992
WTW (mm)	−0.054 ± 0.095	−0.082, −0.027	−0.241, 0.132	0.373
ACD (mm)	0.095 ± 0.042	0.083, 0.107	0.013, 0.177	0.165
LT (mm)	0.110 ± 0.072	0.089, 0.131	−0.033, 0.253	0.285
AL (mm)	−0.036 ± 0.058	−0.054, −0.020	−0.152, 0.079	0.231

SD, standard deviation; CI, confidence interval; LoA, limits of agreement; K, keratometry; CCT, central corneal thickness; WTW, white-to-white distance; ACD, anterior chamber depth; LT, lens thickness; AL, axial length.

## 4 Discussion

A recent review concluded that optical biometers based on SS-OCT technology perform very well when measuring axial length, especially in eyes with advanced cataract (3). The long wavelengths used may play an important role due to their increased ability to penetrate the eye. That study indicated that the devices lead to only small failure rates when measuring axial lengths and, when that was not possible, it was mainly in eyes with a grade of ≥ 4. This is the main reason we considered this as an inclusion criterion to assess how two commercially available SS-OCT biometers performed clinically. To the best of our knowledge there is no data published on whether the Eyestar 900 biometer effectively measures eyes with dense cataracts, nor on its agreement with the Argos device.

### 4.1 Acquisition success rate

Our outcomes showed that the acquisition rate of the Eyestar 900 was the same as the Argos biometer, in both cases 100%. Table 3 was developed to compare our results with those found by other studies using SS-OCT biometers in eyes with dense cataracts. It describes the sample of eyes recruited, the cataract type for the whole sample, and when not measured, and the axial length acquisition success rate for several publications using different SS-OCT biometers. No publications have reported the use of the Eyestar 900 in dense cataracts; it is therefore not possible to directly compare our study with previous ones. For the Argos biometer, Tamaoki et al. (8) reported an acquisition success rate of 89.9% in a sample of 99 eyes with a grade of ≥ 4 according to the Emery-Little classification. The eyes which could not be measured presented mature or white cataracts. In another study (9) including a large sample of eyes (*n* = 213) with cataracts of grades 4, 5 and white, these same authors obtained values of 69.5 and 93.4% using the standard and ERV mode, respectively. They indicated that when the Argos biometer failed to measure the axial length using the standard mode, the ERV mode was utilized. In a previous study carried out by our group (11), we obtained a 100% success rate using this biometer in a sample of 51 eyes with a grade of ≥ 3 and a difunctional lens index of ≤ 5. Despite the fact that in the current study the sample included eyes with a higher mean LOCS III score (4.37 ± 0.68) than that reported by Tañá-Rivero et al. (11) (3.63 ± 0.92), the acquisition rate was 100% in both cohorts, showing that this biometer performs well in eyes with dense cataracts. Other SS-OCT biometers have shown lower rates of acquisition success and varied depending on the study published. Table 3 shows the detailed numbers and type of cataract, when available, in those unsuccessfully measured eyes. For example, for the OA-2000 biometer, Tamaoki et al. (8) reported a lower percentage (80.8%) in eyes with grade ≥ 4 based on the Emery-Little classification, but, in contrast, Vasavada et al. (7) reported a higher percentage (98.4%). Notwithstanding, it should be considered that the sample of these latter authors (*n* = 124) included eyes with low-grade cataracts, which could explain the greater success reported. The percentage obtained with the Anterion biometer was 94.12% for a cataract degree of ≥ 3 and a difunctional lens index of ≤ 5 (11). For the IOLMaster 700, specifically, Hirnschall et al. (5) for example, in a sample of 23 eyes found a value of 91.3%; Henriquez et al. (6) reported a value of 84.4% in 45 eyes; Tamaoki et al. (8, 9) published values of 63.6 and 61.5% in the largest samples published to date, involving 99 and 213 eyes; González-Godínez et al. (10) found a value of 78.57% in 70 eyes; and, finally, Tañá-Rivero et al. (11) indicated a value of 98.04% in 51 eyes. In general, the cataract types that resulted in failed acquisitions were mainly mature or white cataracts. In fact, some authors (10) indicated that the cut-off for SS-OCT biometers may well be up to a subcapsular posterior degree of 4 and a nuclear opalescence degree of 5. As for dense nuclear opacity above the last value indicated and intumescent cataracts, immersion ultrasound biometers remain the best option. It seems that the increasing success rate is related to the use of a longer wavelength (with better signal-to-noise ratio) since shorter wavelengths mean shallower penetration due to scattering (20). In our study, the two biometers use the same, long, wavelength (1,060 nm). OCT sensitivity decreases with depth and, particularly in the case of dense cataracts, due to light energy attenuation making the measurement challenging. Tamaoki et al. (9) indicated that the difficulty of measuring axial length in white cataracts is due to light scattering even when using SS-OCT biometers operating at long wavelengths. These authors reported that axial length measurement was not possible using the Argos biometer for 3 eyes with grade 6 LOCS III scores using the standard mode, but the ERV mode was an excellent option for measuring this parameter in difficult cases. We did not use the ERV mode in our sample and all the measurements were taken using the standard mode. This could be explained by the fact that the majority of the eyes measured showed grades 4 and 5 (89.1%), and only 5 eyes (10.9 %) presented grade 6; in these 5 eyes, it is possible that the opacification did not affect the measurement. We consider that the ERV mode is an excellent option that can be employed when no measurement is possible in cases of dense or white cataracts. In addition, dilating the pupil by means of drugs has also been considered for eyes with dense cataracts when obtaining measurements proves difficult (21). Note that a high number of eyes recruited in a study could explain also the lower acquisition rates of the studies indicated.

**TABLE 3:** Clinical studies that have used different SS-OCT optical biometers and reported axial length acquisition success rates in eyes with dense cataracts.

SS-OCT optical biometer	References	Number of eyes	Acquisition success rates (%)	Cataract type whole sample (eyes)	Cataract type unsuccessfully measured (eyes)
Argos	Tamaoki et al. (8)	99	89.9	§grade ≥ 4	Mature or white cataract (10)
	Tamaoki et al. (9)	213	69.5 93.4 with ERV	§grade 4 (115), grade 5 (65), white cataracts (33)	NA NA
	Tañá-Rivero et al. (11)	51	100	*grade ≥ 3, DLI ≤ 5	
	Current study	46	100	*grade ≥ 4	
IOLMaster 700	Hirnschall et al. (5)	23	91.3	Nuclear cataract (10), PSC (9) and nuclear + PSC (4)	Nuclear cataract (2)
	Henriquez et al. (6)	45	84.4	*NC: 4.76; NO: 4.96; C: 3.91; P: 3.22	*NC: 5.45; NO: 5.27; C: 4.64; P: 4.27
	Tamaoki et al. (8)	99	63.6	§grade ≥ 4	Mature or white cataract (29), grade 4 with PSC (3) and without PSC (3)
	Tamaoki et al. (9)	213	61.5	§grade 4 (115), grade 5 (65), white cataracts (33)	White cataracts (25)
	González-Godínez et al. (10)	70	78.57	*grades ≥ NC 4, NO 4, C 4, and P 3	^‡^Intumescent cataracts
	Tañá-Rivero et al. (11)	51	98.04	*grade ≥ 3, DLI ≤ 5	*NC 6 (1)
OA-2000	Vasavada et al. (7)	124^†^	98.4	§grade 1 (23), grade 2 (30), grade 3 (33), grade 4 (24), grade 5 (14)	NA
	Tamaoki et al. (8)	99	80.8	§grade ≥ 4	Mature or white cataract (17), grade 4 without PSC (1)
Anterion	Tañá-Rivero et al. (11)	51	94.12	*grade ≥ 3, DLI ≤ 5	*PSC 3 (1), NC 4 (1) and NC 6 (1)
Eyestar 900	Current study	46	100	*grade ≥ 4	

Argos (Alcon Labs); IOLMaster 700 (Carl Zeiss Meditec), OA-2000 (Tomey); Anterion (Heidelberg Engineering); Eyestar 900 (Haag-Streit). PSC, posterior subcapsular cataracts; NA, not available; NC, nuclear color;

* for LOCS III; NO, nuclear opalescence; C, cortical; P, subcapsular posterior;

^†^, note that this sample includes low-grade cataracts (see cataract type column); §, Emery–Little classification; ERV, enhanced retina visualization mode,

‡, total failure.

The successful measuring of axial length and its reliability correlate with the good refractive outcomes we obtained 1-month post-surgery (mean spherical equivalent of −0.11 ± 0.42 D with 86.95% of eyes within ± 0.50 D and 97.85% within ± 1.00 D). It is well known that accurate measurements of axial length are extremely important for calculating IOL power. Our results show that there were statistically significant differences in axial length measurements between pre- and post-cataract surgery (*p* < 0.001). However, these differences have no impact on IOL calculation nor, therefore, the refractive accuracy as reported.

### 4.2 Agreement

In relation to the agreement between the two instruments we obtained statistically significant differences for the 7 parameters analyzed (see Table 1 for mean values, SD, ranges and intervals, *p* < 0.001). Figure 1 shows the different Bland-Altman plots created for each parameter and Table 2 presents the agreement values. For K1 and K2 the mean differences were 0.106 and 0.128 D, respectively. Considering the maximum mean difference of 0.128 D this would lead to a difference of about 0.18 D in the IOL power when calculated [a difference of 1.0 D in the K value would cause a difference of about 1.40 D in the IOL power calculation (22)]. This does not impact IOL power choice because of the 0.50 D step in IOL manufacturing. However, the LoA width should be considered as this exceeds 0.50 D (being up to about 0.75 D) and this may indeed affect the choice of IOL power. In relation to CCT, the mean difference was −6.34 μm with a LoA width of 25.99 μm. This value is at the limit of modifying the intraocular pressure measurement since it has been estimated that there is about 1 mm Hg of correction for every 25 μm of deviation from a mean CCT of 550 μm (23). The mean difference reported for WTW measurements was −0.054 ± 0.095 mm and the LoA range was 0.373 mm. The mean difference for ACD was 0.095 mm with a LoA width of 0.165 mm. On average, it has been reported that a 1 mm deviation in ACD could lead to a refractive error of 1.5 D in IOL power (24), meaning that our mean difference and LoA width would not produce any significant change in the IOL power calculation (< 0.25 D). For LT, the differences may also have no clinical impact on the calculation since this was 0.110 mm (LoA: 0.285 mm) and a 0.2 mm increase in LT would change the IOL power by 0.2 D when using the Olsen or Holladay 2 formulas (25, 26). We consider that for both the ACD and LT measurements the two biometers can be used interchangeably. Finally, we obtained a mean axial length difference of −0.036 mm. This value is small, and, taking into account the fact that a 0.1 mm error in axial length would yield a refraction error of about 0.27 D (25), the differences between the two biometers would not affect the IOL power calculation and, therefore, they can be used interchangeably for axial length measurements. However, as we have done for the other parameters analyzed, we also have to consider the LoA width (0.231 mm), which surpasses the limits considered clinically negligible (0.62 D), and should be taken into account in the IOL power calculation.

Tamaoki et al. (9) compared pre- and postoperative axial length measurements and found median absolute differences of 0.05 and 0.08 mm for the Argos biometer (without using and using the ERV mode), respectively. Our mean difference for the Argos (without the ERV mode) was better than that reported by these authors (0.020 ± 0.029 mm), and the value for the Eyestar 900 was higher (0.036 ± 0.034 mm, see Figure 2). In both cases and considering the LoA width (0.115 mm for the Argos and 0.134 mm for the Eyestar 900) the pre- and post-surgery differences can be considered clinically negligible and would not affect the IOL power calculation [based on the fact that a 0.1 mm error would yield a refraction error of about 0.27 D (25)]. The accuracy of the surgery reveals that 86.95% of the eyes were within ± 0.50 D. The difference between the pre- and post-surgery axial length was ≤ 0.1 mm in 97.82% of eyes using the Eyestar 900 and in 100% of eyes with the Argos; Tamaoki et al. (9) reported a percentage of 58.8% of eyes for the same difference. Differences between the samples could be the reason for the better outcomes in our cohort. Note that they assessed 213 eyes where 33 presented white cataracts and other ocular diseases other than cataracts that may have affected the measurement.

Our clinical study shows that SS-OCT biometry can be used to successfully measure axial length in cases of dense cataracts and improves refractive outcomes since reliable measurements are used for the IOL power calculation. We consider that SS-OCT biometry may be used in eyes with dense cataracts. The differences between the two biometers in some parameters may have a clinically significant impact but should be judged individually. Furthermore, axial length differences pre- and post-surgery can be considered clinically negligible and should not affect the IOL power calculation. Limitations of our study are the number of eyes recruited, and the lacking short and long eyes. Future studies with larger samples of dense cataracts, including white cataracts and eyes with long and short axial lengths should be undertaken to fully analyze the performance of SS-OCT biometry.

## Data Availability

The original contributions presented in the study are included in the article/supplementary material, further inquiries can be directed to the corresponding author.

## References

[1] McAlinden C Wang Q Pesudovs K Yang X Bao F Yu A Axial length measurement failure rates with the IOLMaster and Lenstar LS 900 in eyes with cataract. *PLoS One.* (2015) 10:e0128929. 10.1371/journal.pone.0128929 26061554 PMC4462579

[2] Shammas H Wetterwald N Potvin R. New mode for measuring axial length with an optical low-coherence reflectometer in eyes with dense cataract. *J Cataract Refract Surg.* (2015) 41:1365–9. 10.1016/j.jcrs.2014.10.032 26210047

[3] Tañá-Rivero P Tañá-Sanz S Pastor-Pascual F Ruiz-Mesa R Montés-Micó R. Axial length measurement failure rates using optical biometry based on swept-source OCT in cataractous eyes. *Expert Rev Med Devices.* (2022) 19:633–40. 36062739 10.1080/17434440.2022.2118047

[4] Tañá-Rivero P Aguilar-Córcoles S Tello-Elordi C Pastor-Pascual F Montés-Micó R. Agreement between 2 swept-source OCT biometers and a Scheimpflug partial coherence interferometer. *J Cataract Refract Surg.* (2021) 47:488–95. 10.1097/j.jcrs.0000000000000483 33252569

[5] Hirnschall N Varsits R Doeller B Findl O. Enhanced penetration for axial length measurement of eyes with dense cataracts using swept source optical coherence tomography: A consecutive observational study. *Ophthalmol Ther.* (2018) 7:119–24. 10.1007/s40123-018-0122-1 29498015 PMC5997603

[6] Henriquez M Zúñiga R Camino M Camargo J Ruiz-Montenegro K Izquierdo L Jr. Effectiveness and agreement of 3 optical biometers in measuring axial length in the eyes of patients with mature cataracts. *J Cataract Refract Surg.* (2020) 46:1222–8. 10.1097/j.jcrs.0000000000000237 32379086

[7] Vasavada S Patel P Vaishnav V Ashena Z Srivastava S Vasavada V Comparison of optical low-coherence reflectometry and sweptsource OCT-based biometry devices in dense cataracts. *J Refract Surg.* (2020) 36:557–64. 10.3928/1081597X-20200612-03 32785730

[8] Tamaoki A Kojima T Hasegawa A Yamamoto M Kaga T Tanaka K Clinical evaluation of a new swept-source optical coherence biometer that uses individual refractive indices to measure axial length in cataract patients. *Ophthalmic Res.* (2019) 62:11–23. 10.1159/000496690 30889604

[9] Tamaoki A Kojima T Hasegawa A Yamamoto M Kaga T Tanaka K Evaluation of axial length measurement using enhanced retina visualization mode of the swept-source optical coherence tomography biometer in dense cataract. *Ophthalmic Res.* (2021) 64:595–603. 10.1159/000515054 33550307

[10] González-Godínez S Saucedo-Urdapilleta R Mayorquín-Ruiz M Velasco Barona C Moragrega-Adame E Domínguez-Varela I Ocular biometry in dense cataracts: Comparison of partial-coherence interferometry, swept-source optical coherence tomography and immersion ultrasound. *Indian J Ophthalmol.* (2022) 70:107–11. 10.4103/ijo.IJO_854_21 34937218 PMC8917608

[11] Tañá-Rivero P Aguilar-Córcoles S Tañá-Sanz P Tañá-Sanz S Montés-Micó R. Axial length acquisition success rates and agreement of four optical biometers and one ultrasound biometer in eyes with dense cataracts. *Eye Vis (Lond).* (2023) 10:35. 10.1186/s40662-023-00352-3 37653460 PMC10472586

[12] Sorkin N Achiron A Abumanhal M Abulafia A Cohen E Gutfreund S Comparison of two new integrated SS-OCT tomography and biometry devices. *J Cataract Refract Surg.* (2022) 48:1277–84. 10.1097/j.jcrs.0000000000000974 35608316

[13] Lender R Mirsky D Greenberger R Boim Z Ben-Yaakov L Kashtan C Evaluation of three biometric devices: Ocular parameters and calculated intraocular lens power. *Sci Rep.* (2022) 12:19478. 10.1038/s41598-022-24017-8 PMC9663510 36376354

[14] Galzignato A Lupardi E Hoffer K Barboni P Schiano-Lomoriello D Savini G. Repeatability of new optical biometer and agreement with 2 validated optical biometers, all based on SS-OCT. *J Cataract Refract Surg.* (2023) 49:5–10. 10.1097/j.jcrs.0000000000001023 36026703

[15] Sorkin N Zadok T Barrett G Chasid O Abulafia A. Comparison of biometry measurements and intraocular lens power prediction between 2 SS-OCT-based biometers. *J Cataract Refract Surg.* (2023) 49:460–6. 10.1097/j.jcrs.0000000000001146 36719441

[16] Domínguez-Vicent A Venkataraman A Dalin A Brautaset R Montés-Micó R. Repeatability of a fully automated swept-source optical coherence tomography biometer and agreement with a low coherence reflectometry biometer. *Eye Vis (Lond).* (2023) 10:24. 10.1186/s40662-023-00343-4 37264436 PMC10236819

[17] Bograd A Himmel I Pfister I Seiler T Frueh B Tappeiner C. Comparison of corneal measurements in keratoconus eyes with two swept-source-optical coherence tomography devices and a Scheimpflug device. *Graefes Arch Clin Exp Ophthalmol.* (2024) 262:891–901. 37688609 10.1007/s00417-023-06219-6

[18] Chylack L Jr. Wolfe J Singer D Leske M Bullimore M Bailey I The lens opacities classification system III. *Arch Ophthalmol.* (1993) 111:831–6. 8512486 10.1001/archopht.1993.01090060119035

[19] Gioia M De Bernardo M Pagliarulo S Cione F Mottola F La Marca A Evaluation of tropicamide-phenylephrine mydriatic eye drop instillation on choroidal thickness. *J Clin Med.* (2023) 12:6355. 10.3390/jcm12196355 37834998 PMC10573589

[20] Povazay B Hermann B Unterhuber A Hofer B Sattmann H Zeiler F Three-dimensional optical coherence tomography at 1050 nm versus 800 nm in retinal pathologies: Enhanced performance and choroida penetration in cataract patients. *J Biomed Opt.* (2007) 12:041211. 10.1117/1.2773728 17867800

[21] Bettach E Weill Y Aronovitz Y Zadok D Gelman E Abulafia A. Advantageous effect of pupil dilation on the quality of optical biometry axial length measurement in individuals with dense cataract. *J Cataract Refract Surg.* (2022) 48:1248–52. 10.1097/j.jcrs.0000000000000964 35514045

[22] Hua Y Qiu W Xiao Q Wu Q. Precision (repeatability and reproducibility) of ocular parameters obtained by the Tomey OA-2000 biometer compared to the IOLMaster in healthy eyes. *PLoS One.* (2018) 13:e0193023. 10.1371/journal.pone.0193023 29486009 PMC5828443

[23] Kohlhaas M Boehm A Spoerl E Pürsten A Grein H Pillunat L. Effect of central corneal thickness, corneal curvature, and axial length on applanation tonometry. *Arch Ophthalmol.* (2006) 124:471–6. 16606871 10.1001/archopht.124.4.471

[24] Olsen T. Calculation of intraocular lens power: A review. *Acta Ophthalmol Scand.* (2007) 85:472–85. 10.1111/j.1600-0420.2007.00879.x 17403024

[25] Olsen T Hoffmann P. C constant: New concept for ray tracing– assisted intraocular lens power calculation. *J Cataract Refract Surg.* (2014) 40:764–73. 10.1016/j.jcrs.2013.10.037 24767910

[26] Shammas H Ortiz S Shammas M Kim S Chong C. Biometry measurements using a new large-coherence-length swept-source optical coherence tomographer. *J Cataract Refract Surg.* (2016) 42:50–61. 10.1016/j.jcrs.2015.07.042 26948778

